# Author Correction: Histone acetylome-wide associations in immune cells from individuals with active *Mycobacterium tuberculosis* infection

**DOI:** 10.1038/s41564-022-01236-3

**Published:** 2022-09-02

**Authors:** Ricardo C. H. del Rosario, Jeremie Poschmann, Carey Lim, Catherine Y. Cheng, Pavanish Kumar, Catherine Riou, Seow Theng Ong, Sherif Gerges, Hajira Shreen Hajan, Dilip Kumar, Mardiana Marzuki, Xiaohua Lu, Andrea Lee, Giovani Claresta Wijaya, Nirmala Arul Rayan, Zhong Zhuang, Elsa Du Bruyn, Cynthia Bin Eng Chee, Bernett Lee, Josephine Lum, Francesca Zolezzi, Michael Poidinger, Olaf Rotzschke, Chiea Chuen Khor, Robert J. Wilkinson, Yee T. Wang, George K Chandy, Gennaro De Libero, Amit Singhal, Shyam Prabhakar

**Affiliations:** 1 Genome Institute of Singapore, Agency for Science, Technology and Research (A*STAR), Singapore, Singapore; 2 Stanley Center for Psychiatric Research, Broad Institute of MIT and Harvard, Cambridge, MA USA; 3 Department of Genetics, Harvard Medical School, Boston, MA USA; 4 Inserm, Université de Nantes, Centre de Recherche en Transplantation et Immunologie, ITUN, Nantes, France; 5 Singapore Immunology Network, A*STAR, Singapore, Singapore; 6 A*STAR Infectious Diseases Labs, A*STAR, Singapore, Singapore; 7 Wellcome Centre for Infectious Diseases Research in Africa, University of Cape Town, Cape Town, South Africa; 8 Institute of Infectious Disease and Molecular Medicine, University of Cape Town, Cape Town, South Africa; 9 Division of Medical Virology, Department of Pathology, University of Cape Town, Cape Town, South Africa; 10 Lee Kong Chian School of Medicine, Nanyang Technological University, Singapore, Singapore; 11 Analytic and Translational Genetics Unit, Department of Medicine, Massachusetts General Hospital, Boston, MA USA; 12 Tuberculosis Control Unit, Tan Tock Seng Hospital, Singapore, Singapore; 13 Department of Infectious Disease, Imperial College, London, UK; 14 The Francis Crick Institute, London, UK; 15 Department of Biomedicine, University of Basel, Basel, Switzerland

**Keywords:** Tuberculosis, Functional genomics, Tuberculosis

Correction to: *Nature Microbiology* 10.1038/s41564-021-01049-w, published online 31 January 2022.

This paper was originally published under a standard Springer Nature license (© The Author(s), under exclusive licence to Springer Nature America, Inc.). It is now available as an Open Access paper under a Creative Commons Attribution 4.0 International license, © The Author(s). The error has been corrected in the HTML and PDF versions of the article.

