# Supplementary material for: Histone acetylome-wide associations in immune cells from individuals with active Mycobacterium tuberculosis infection
Source: Nat Microbiol. 2022 Jan 31;7(2):312–26. doi: 10.1038/s41564-021-01049-w (PMC9439955; doi:10.1038/s41564-021-01049-w)
Supplement: Supplementary file 1 — Supplementary Methods, Tables 1,14,18,32,35–37 and Figs. 1–9. [file 41564_2021_1049_MOESM1_ESM.pdf]

nature portfolio

https://doi.org/10.1038/s41564-021-01049-w

Supplementary information

Histone acetylome-wide associations in immune cells from individuals with active *Mycobacterium tuberculosis* infection

In the format provided by the authors and unedited

Supplementary Information

Histone acetylome-wide associations in immune cells from individuals with active *Mycobacterium tuberculosis* infection

Ricardo C.H. del Rosario[1,2,3,†], Jeremie Poschmann[1,4,†], Carey Lim[5,6#], Catherine Y. Cheng[5#], Pavanish Kumar[5], Catherine Riou[7,8,9], Seow Theng Ong[10], Sherif Gerges[2,3,11], Hajira Shreen Hajan[1], Dilip Kumar[4], Mardiana Marzuki[5,6], Xiaohua Lu[5], Andrea Lee[5,6], Giovani Claresta Wijaya[1], Nirmala Arul Rayan[1], Zhong Zhuang[10], Elsa Du Bruyn[7,8], Cynthia Bin Eng Chee[12], Bernett Lee[5], Josephine Lum[5], Francesca Zolezzi[5], Michael Poidinger[5], Olaf Rotzschke[5], Chiea Chuen Khor[1], Robert J. Wilkinson[7,8,13,14], Yee T. Wang[12], K. George Chandy[10], Gennaro De Libero[5,15], Amit Singhal[5,6,10,*], Shyam Prabhakar[1,*]

[1]Genome Institute of Singapore, Agency for Science, Technology and Research (A*STAR), Singapore, 138672.
[2]Stanley Center for Psychiatric Research, Broad Institute of MIT and Harvard, 75 Ames St., Cambridge MA 02144.
[3]Department of Genetics, Harvard Medical School. Boston, Massachusetts, USA.
[4]Inserm, Université de Nantes, Centre de Recherche en Transplantation et Immunologie, UMR1064, ITUN, 44000 Nantes, France.
[5]Singapore Immunology Network, A*STAR, Singapore 138648.
[6]A*STAR Infectious Diseases Labs, A*STAR, Singapore 138648.
[7]Wellcome Centre for Infectious Diseases Research in Africa, University of Cape Town; Observatory 7925, South Africa.
[8]Institute of Infectious Disease and Molecular Medicine, University of Cape Town; Observatory 7925, South Africa.
[9]Division of Medical Virology, Department of Pathology; University of Cape Town; Observatory 7925, South Africa.
[10]Lee Kong Chian School of Medicine, Nanyang Technological University Singapore, Singapore 636921.
[11]Analytic and Translational Genetics Unit, Department of Medicine, Massachusetts General Hospital, Boston, Massachusetts, USA.
[12]Tuberculosis control unit, Tan Tock Seng Hospital, Singapore 308087.
[13]Department of Infectious Disease, Imperial College, London W12 ONN, United Kingdom
[14]The Francis Crick Institute, London, United Kingdom
[15]Department of Biomedicine, University of Basel, 4056 Basel, Switzerland.

†Equal contribution
[#] Equal contribution
*Correspondence to: prabhakars@gis.a-star.edu.sg, amit_singhal@idlabs.a-star.edu.sg

This Supplementary Information file contains the following:

- Supplementary Methods pages 2-4
- References for Supplementary Methods page 4
- Supplementary Tables 1,14,18,32,35,36,37 pages 5-8
- Legends of all Supplementary Tables in the Excel File pages 9-10
- Supplementary Figures 1-9 pages11-16

Supplementary Methods

SNP Calling. All discovery samples indicated in Supplementary Table 1 were used for SNP calling as described previously[1,2]. Following the genome analysis toolkit (GATK) best practices, the BAM file for each ChIP-seq sample was realigned at given indel sites and base qualities were then recalibrated[3]. For each sample, GATK's Haplotype Caller was run in reference confidence model (GVCF) mode, and subsequently multi-sample SNP calling was performed on all samples (granulocytes plus monocytes) using GATK's Genotype GVCFs tool. Low-confidence SNP calls were filtered by applying thresholds to GATK parameter values. We filtered out SNPs if they satisfied any of the following: QD<4.185, Inbreeding Coefficient<-0.398, within a SNP cluster of more than 19 SNPs in a 100bp window, Homopolymer run≥18, and Hardy-Weinberg equilibrium binomial test *P*-value≤1e-3. We additionally imposed the criterion that a SNP should be supported by at least 5 non-reference reads across all datasets, and at least one of the datasets should have 3 or more non-reference reads. We also discarded SNPs contained within UCSC Genome Browser self-chains blocks with a normalized score>90. Based on this pipeline, we discovered 362,021 SNPs in ChIP-seq peaks, of which 247,411 were polymorphic in the granulocyte dataset and 273,753 in the monocyte dataset.

Genotyping. All samples were genotyped using the Illumina OmniExpress v1.1 Beadarray, which assesses >700,000 representative genetic markers in a genome-wide manner. A subset of SNPs was further validated using the Sequenom MassArray primer extension platform. Both methods have been used extensively by others and us and have been found to result in >99.99% concordance[4]. Genotypes inferred were compared against genotype calls from ChIP-seq reads to identify and correct sample swaps in the ChIP-seq pipeline.

Histone Acetylation QTLs. We used the genotype-independent signal correlation and imbalance (G-SCI) test to call haQTLs in granulocytes and monocytes[1]. Only high-quality samples from the ethnically homogeneous discovery cohort were used in this analysis (46 granulocyte samples, 32 monocyte samples). The G-SCI test does not require prior knowledge of genotypes. Rather, it infers genotype likelihoods from ChIP-seq read counts and then integrates over all possible genotypes to calculate the statistical significance of an haQTL. This test combines information from peak height vs. genotype correlation across samples and allelic imbalance in read count within heterozygous individuals. These two signatures of an haQTL are combined into a single *P*-value. To infer haQTLs independently of disease state, disease status was regressed out from the peak-height matrix used for DA peak calling, and the residual was used for G-SCI analysis[2]. In this study, the G-SCI test procedure was refined in two ways. Firstly, for each SNP k, we replaced the uniform allele frequency prior used in the original study with a SNP-specific allele frequency f_k. Thus, for each SNP, we estimated four parameters by maximum-likelihood (f_k, α, β, and σ) instead of three. Secondly, we modified the method for permutation testing to convert raw *P*-values to final *P*-values. The original permutation approach[1] randomized the data by permuting peak heights across samples for the same SNP. In addition, reference and non-reference base calls were

randomly flipped with probability 0.5 at heterozygous sites. In the current study, this procedure was generalized and accelerated by randomly swapping data between ChIP-seq units. A unit was defined as the peak height and allelic counts for a SNP in a single individual. Thus, the total number of units would equal the number of SNPs times the number of individuals. Units were then binned in three dimensions according to their peak height, number of reads covering the SNP and fraction of non-reference reads. Units within the same three-dimensional bin were randomly permuted, and the haQTL *P*-value was then calculated for each SNP. As before, the permutation-test *P*-value was then calculated based on the rank of the raw *P*-value relative to the *P*-values of permuted data. Again, as before, SNPs with an adjusted *P*-value of 0 after 1 million permutations were assigned an adjusted *P*-value of 5e-7. FDR correction and filtering for QTL effect size was performed as before[1].

Overlap between haQTLs and eQTLs. We downloaded the granulocyte eQTLs[5] and obtained the subset that was in tight linkage with SNPs we called within granulocyte ChIP-seq peaks. The genotypes from the European population of 1000 Genomes were used in the LD calculation, with a threshold of $R^2 \geq 0.8$ and maximum distance of 500 Kb. As before, we corrected for LD within the eQTL set by constructing the LD network of the eQTL set and replaced each connected component in the network by the SNP with the best eQTL *P*-value[1]. This resulted in 1,257 Naranbhai eQTLs used for analysis. We downloaded the naïve monocyte eQTLs[6] and used the same procedure to obtain a final set of 2,216 eQTLs for further analysis. We also downloaded neutrophil and monocyte eQTLs[7] and retained those with an FDR *Q*-value≤0.05. If a gene had more than one eQTL, the one with the best *P*-value was kept and the others were discarded. Chen et al. eQTLs were further filtered based on LD as described above, resulting in 3,069 neutrophil and 3,460 monocyte eQTLs for further analysis. Note that the vast majority of granulocytes in peripheral blood are neutrophils. For each SNP from the four eQTL sets, the LD with haQTLs from the corresponding cell type was calculated using the same 1000 Genome population and LD thresholds as above. To calculate the statistical significance of the LD, we randomly chose 1.5 million SNPs as a control set, and used a method as before wherein the likelihood of linkage of the control set and eQTL set with haQTLs was calculated while adjusting for allele frequencies, genomic location biases (distance from nearest TSS of a gene), biases in LD block sizes between the eQTL SNP and control SNP, and correcting for LD within each of the four eQTL sets[1].

Overlap with published haQTL datasets. We downloaded neutrophil and monocyte H3K27 haQTLs[7] and processed them in a similar manner to eQTLs. First, we filtered for FDR *Q*-value≤0.05 and retained only the most significant haQTL for each ChIP-seq peak. We then discarded haQTLs that were not in LD with any SNP within our ChIP-seq peaks (LD was defined as $R^2 \geq 0.8$, distance≤500kb). As above, these were further filtered to retain only one haQTL per LD cluster, resulting in 3,625 neutrophil H3K27ac QTLs and 4,357 monocyte H3K27ac QTLs. We then calculated the statistical significance of the linkage of these haQTLs with our own haQTLs using the method described above for eQTLs.

Partitioned Heritability. Using the GWAS results from Luo and colleagues[8], we performed linkage-disequilibrium (LD) score regression[9] to quantify proportion of heritability of both differentially and non-differentially acetylated peaks within peripheral blood monocytes and granulocytes. For all acetylated sites described earlier, we created annotations by extending sites by 500 bp at both ends. We then partitioned heritability by functional category using the full BaselineLD v2.2 model containing 83 functional annotations (using the --h2 flag) and the 1000 Genomes Project Phase 3 East Asian reference haplotypes[10]. We excluded variants in the HLA region (chr6:26 Mb-34 Mb). All peripheral LDSC files used in this analysis are available at (https://data.broadinstitute.org/alkesgroup/LDSCORE/).

References for Supplementary Methods

1. del Rosario, R. C.-H. *et al.* Sensitive detection of chromatin-altering polymorphisms reveals autoimmune disease mechanisms. *Nat. Methods* **12**, 458–464 (2015).
2. Sun, W. *et al.* Histone Acetylome-wide Association Study of Autism Spectrum Disorder. *Cell* **167**, 1385-1397.e11 (2016).
3. DePristo, M. A. *et al.* A framework for variation discovery and genotyping using next-generation DNA sequencing data. *Nat. Genet.* **43**, 491–498 (2011).
4. Aung, T. *et al.* Genetic association study of exfoliation syndrome identifies a protective rare variant at *LOXL1* and five new susceptibility loci. *Nat. Genet.* **49**, 993–1004 (2017).
5. Naranbhai, V. *et al.* Genomic modulators of gene expression in human neutrophils. *Nat. Commun.* **6**, 7545 (2015).
6. Fairfax, B. P. *et al.* Innate immune activity conditions the effect of regulatory variants upon monocyte gene expression. *Science* **343**, 1246949 (2014).
7. Chen, L. *et al.* Genetic Drivers of Epigenetic and Transcriptional Variation in Human Immune Cells. *Cell* **167**, 1398-1414.e24 (2016).
8. Luo, Y. *et al.* Early progression to active tuberculosis is a highly heritable trait driven by 3q23 in Peruvians. *Nat. Commun.* **10**, 3765 (2019).
9. Finucane, H. K. *et al.* Partitioning heritability by functional annotation using genome-wide association summary statistics. *Nat. Genet.* **47**, 1228–1235 (2015).
10. 1000 Genomes Project Consortium *et al.* A global reference for human genetic variation. *Nature* **526**, 68–74 (2015).

Supplementary Tables

Supplementary Table 1. Number of high-quality granulocyte and monocyte ChIP-seq datasets from discovery and validation cohorts remaining after QC. Sizes of corresponding final core sets are indicated in parentheses. Number of consensus ChIP-seq peaks in high-quality datasets. Number of differentially acetylated (DA) peaks in core set.

Singapore ChIP-seq	ATB Individuals	HC Individuals	ChIP-seq peaks	DA peaks
Discovery granulocytes	23 (16)	23 (20)	12264	2065
Validation granulocytes	14 (12)	14 (14)	16118	2367
Discovery monocytes	16 (11)	16 (16)	16561	2144
Validation monocytes	8 (8)	21 (21)	22534	1848
Total ChIP-seq datasets	61 (47)	74 (71)		

Supplementary Table 14. Number of high-quality granulocyte and monocyte RNA-seq datasets remaining after QC. Sizes of corresponding final core sets are indicated in parentheses. Number of differentially expressed genes (DE) in core set.

RNA-seq	ATB Individuals	HC Individuals	DE genes
Granulocytes	19 (18)	19 (18)	1979
Monocytes	20 (17)	19 (19)	1869
Total RNA-seq datasets	39 (35)	38 (37)	

Supplementary Table 18. Pearson correlation of log-fold-change between DE genes and their associated DA peaks. Correlations were calculated for DE and DA sets defined using the default FDR threshold (FDR≤0.05), and also a more stringent threshold (FDR≤0.01). The number of DE genes with at least one associated with DA peak and the total number of points used in the correlation are shown in parentheses. *P*-values indicate concordance of the fold-change direction, calculated using the hypergeometric test.

	Correlation (FDR 0.10)	*P*-value	Correlation (FDR 0.05)	*P*-value
Granulocytes	0.71 (275, 303)	1.14e-41	0.73 (214, 233)	2.54e-33
Monocytes	0.30 (139, 148)	3.11e-3	0.38 (72,74)	2.86e-2

Supplementary Table 32. Enrichment of DA peaks (relative to all peaks) near individual genes. The most significantly enriched genes are shown for each DA peak set. The last column indicates whether the DA peak cluster overlaps an SE by at least one peak.

Peak Set	Gene	Rank	*P*-value	FDR *Q*-value	Observed	Expected	Enrichment	Overlaps SE
Discovery granulocytes up	*FNDC1*	1	6.78e-07	1.80e-03	7	0.71	9.9	Yes
	TAGAP	2	3.14e-06	4.17e-03	8	1.06	7.5	No
	HIATL2	3	5.24e-05	3.47e-02	5	0.53	9.4	No
	ZNF782	4	5.24e-05	3.47e-02	5	0.53	9.4	No
	ERLIN1	5	9.62e-05	4.25e-02	4	0.35	11.3	No
Discovery granulocytes down	*SGK1*	1	2.16e-06	5.75e-03	7	0.8	8.8	Yes
	THSD7B	2	1.89e-05	1.71e-02	6	0.72	8.4	Yes
	SLC2A12	3	1.93e-05	1.71e-02	5	0.48	10.4	Yes
Discovery monocytes up	*KCNJ15*	1	3.24e-07	1.24e-03	8	0.81	9.9	Yes
	F2RL2	2	3.91e-06	7.47e-03	6	0.54	11.1	Yes
	RUNX1	3	1.13e-05	1.41e-02	8	1.14	7	No
	ERG	4	2.52e-05	1.41e-02	7	0.94	7.4	No
	GCNT1	5	2.57e-05	1.41e-02	6	0.67	8.9	No
Discovery monocytes down	*AGPAT4*	1	1.47e-08	5.64e-05	9	0.8	11.2	No
	AMZ1	2	4.48e-08	5.71e-05	7	0.49	14.2	Yes
	GNA12	3	4.48e-08	5.71e-05	7	0.49	14.2	Yes
	PRPF18	4	2.22e-06	2.12e-03	6	0.49	12.1	No
	LAT	5	3.49e-06	2.23e-03	7	0.74	9.4	Yes

Supplementary Table 35. Discovery cohort (Cohort 1): association of DA peaks with super-enhancers, relative to all peaks. Enrichment *P*-values: Fisher's exact test, two sided.

	Granulocyte DA peaks	Granulocyte all peaks	Monocyte DA peaks	Monocyte all peaks
No. of peaks in SE	488	2,860	391	3,345
No. of peaks outside SE	1,577	9,404	1,753	13,216
Total Peaks	2,065	12,264	2,144	16,561
Odds Ratio	1.01		0.90	
P-value	0.75		0.03	

Supplementary Table 36. Transcriptomic data sets used for *KCNJ15* differential expression analysis (Fig. 5a).

ID	Country of cohort	Healthy controls	Active TB	Post-treatment active TB	Study reference	Microarray/ RNA-seq platform	GEO accession	Sample type
**UK '10 test set**	United Kingdom	12	21	-	Berry et al., 2010	Illumina Human HT-12 v3.0	GSE19444	Whole blood
**UK '10 training set**	United Kingdom	12	13	-	Berry et al., 2010	Illumina Human HT-12 v3.0	GSE19439	Whole blood
**TG '11**	The Gambia	37	46	-	Maertzdorf et al., 2011	Illumina Human HT-12 v4.0	GSE28623	Whole blood
**UK '10 treatment set**	United Kingdom	-	12	7	Berry et al., 2010	Illumina Human HT-12 v4.0	GSE19435	Whole blood
**Singapore monocytes**	Singapore	19	20	-	This study	RNA-seq Illumina HiSeq 2000	This study	monocytes
**Singapore granulocytes**	Singapore	18	20	-	This study	RNA-seq Illumina HiSeq 2000	This study	granulocytes

Supplementary Table 37. Differentially expressed genes in primary monocytes that over-express *KCNJ15* (KCNJ15^{++}) compared to respective control monocytes. N=5 donors. Outcome of paired two-sided Wald test in DESeq2 package (FDR<0.05) analysis has been depicted.

Gene Name	log2 (fold-change)	FDR *Q*-value	Relation to Apoptosis	References (PMID)
KCNJ15	7.03	5.51E-97		
FANCI	3.20	9.31E-06	Pro-apoptotic	27097374
PRDM15	2.40	1.04E-04	Pro-apoptotic (Genes of this family are pro-apototic)	28476379
MGC27382	-3.33	3.16E-04		
CCND1	-3.14	3.16E-04	Anti-apoptotic	21912938
TMEM128	-3.29	4.54E-04		
PRSS23	2.95	7.83E-04	Anti-apoptotic	30769097
FAM126B	1.78	1.13E-03		
FSTL1	-3.01	2.81E-03	Anti-apoptotic	25139876, 22929303
REXO5	-2.88	3.01E-03		
ADCY6	-2.86	3.01E-03		
IGF2BP1	-2.63	3.01E-03	Anti-apoptotic	25889892
CENPJ	-2.42	3.38E-03	Anti-apoptotic	30626697
SGF29	1.78	3.38E-03		
GOLGB1	-1.74	4.88E-03		
SOCS7	-1.46	6.82E-03	Anti-apoptotic	23392170
EPN1	1.90	6.83E-03	Pro-apoptotic	30595089
CCDC180	2.79	7.99E-03	Pro-apoptotic	30790560
BRD1	1.64	1.03E-02		
PPP1R9A	-2.77	1.35E-02	Anti-apoptotic	15864305
UMPS	-2.17	1.82E-02		
MRPS23	-1.97	3.86E-02	Anti-apoptotic	29069745
SRD5A1	1.88	4.11E-02		
USP37	1.68	4.31E-02	Anti-apoptotic	30482232
OSBPL3	-1.62	4.78E-02	Anti-apoptotic	31509750

Legends of Supplementary Tables in Excel File

Supplementary Table 2. Metadata of ChIP-seq granulocyte discovery cohort
Supplementary Table 3. List of DA peaks for the granulocyte discovery cohort (ChIP-seq)
Supplementary Table 4. Metadata of ChIP-seq granulocyte validation cohort
Supplementary Table 5. List of DA peaks for the granulocyte validation cohort (ChIP-seq)
Supplementary Table 6. Metadata of ChIP-seq monocyte discovery cohort
Supplementary Table 7. List of DA peaks for the monocyte discovery cohort (ChIP-seq)
Supplementary Table 8. Metadata of ChIP-seq monocyte validation cohort
Supplementary Table 9. List of DA peaks for the monocyte validation cohort (ChIP-seq)
Supplementary Table 10. Input corrected and GC corrected peakheights for each granulocyte discovery sample (ChIP-seq)
Supplementary Table 11. Input corrected and GC corrected peakheights for each monocyte discovery sample (ChIP-seq)
Supplementary Table 12. Input corrected and GC corrected peakheights for each granulocyte validation sample (ChIP-seq)
Supplementary Table 13. Input corrected and GC corrected peakheights for each monocyte validation sample (ChIP-seq)
Supplementary Table 15. Metadata of RNA-seq samples
Supplementary Table 16. Differentially expressed genes from monocyte RNA-seq
Supplementary Table 17. Differentially expressed genes from granulocyte RNA-seq
Supplementary Table 19. Metadata of ChIP-seq monocyte SA cohort
Supplementary Table 20. Gene ontology (GREAT) results of DA peak enrichment, granulocytes, discovery cohort, Up DA peaks (see http://great.stanford.edu/public/html/ for detailed description of the columns in the worksheet)
Supplementary Table 21. Gene ontology (GREAT) results of DA peak enrichment, granulocytes, discovery cohort, Down DA peaks (see http://great.stanford.edu/public/html/ for detailed description of the columns in the worksheet)
Supplementary Table 22. Gene ontology (GREAT) results of DA peak enrichment, monocytes, discovery cohort, Up DA peaks (see http://great.stanford.edu/public/html/ for detailed description of the columns in the worksheet)
Supplementary Table 23. Gene ontology (GREAT) results of DA peak enrichment, monocytes, discovery cohort, Down DA peaks (see http://great.stanford.edu/public/html/ for detailed description of the columns in the worksheet)
Supplementary Table 24. Motif enrichment analysis of granulocyte Up DA peaks, discovery cohort
Supplementary Table 25. Motif enrichment analysis of granulocyte Down DA peaks, discovery cohort
Supplementary Table 26. Motif enrichment analysis of monocyte Up DA peaks, discovery cohort
Supplementary Table 27. Motif enrichment analysis of monocyte Down DA peaks, discovery cohort
Supplementary Table 28. Summary of motif enrichment analysis results
Supplementary Table 29. Gorilla analysis of DE genes, granulocytes, RNA-seq cohort, Up DE genes (see http://cbl-gorilla.cs.technion.ac.il/ for detailed description of the columns in the worksheet)

Supplementary Table 30. Gorilla analysis of DE genes, granulocytes, RNA-seq cohort, Down DE genes (see http://cbl-gorilla.cs.technion.ac.il/ for detailed description of the columns in the worksheet)
Supplementary Table 31. Gorilla analysis of DE genes, monocytes, RNA-seq cohort, Up DE genes (see http://cbl-gorilla.cs.technion.ac.il/ for detailed description of the columns in the worksheet)
Supplementary Table 33. Homer output for Super Enhancer analysis of monocyte discovery cohort
Supplementary Table 34. Homer output for Super Enhancer analysis of granulocyte discovery cohort
Supplementary Table 38. list of granulocyte haQTLs identified in this study
Supplementary Table 39. list of monocyte haQTLs identified in this study
Supplementary Table 40. Chen monocyte H3K27ac QTLs in LD with monocyte haQTLs from this study. The list includes all SNPs without collapsing by LD.
Supplementary Table 41. Chen monocyte eQTLs in LD with monocyte haQTLs from this study, The list includes all SNPs without collapsing by LD.
Supplementary Table 42. Fairfax monocyte eQTLs in LD with monocyte haQTLs from this study. The list includes all SNPs without collapsing by LD.
Supplementary Table 43. Chen granulocyte H3K27ac QTLs in LD with granulocyte haQTLs from this study. The list includes all SNPs without collapsing by LD.
Supplementary Table 44. Naranbhai granulocyte eQTLs in LD with granulocyte haQTLs from this study. The list includes all SNPs without collapsing by LD.
Supplementary Table 45. Chen granulocyte eQTLs in LD with granulocyte haQTLs from this study. The list includes all SNPs without collapsing by LD.
Supplementary Table 46. TB GWAS SNPs used in this study
Supplementary Table 47. Leprosy GWAS SNPs used in this study
Supplementary Table 48. Leprosy GWAS SNPs that are in LD with haQTLs. The list includes all SNPs without collapsing by LD.
Supplementary Table 49. Inflammation GWAS SNPs used in this study
Supplementary Table 50. Inflammation GWAS SNPs that are in LD with haQTLs. The list includes all SNPs without collapsing by LD.
Supplementary Table 51. Autoimmune GWAS SNPs used in this study
Supplementary Table 52. Autoimmune GWAS SNPs that are in LD with haQTLs. The list includes all SNPs without collapsing by LD.
Supplementary Table 53. No. of GWAS SNPS that are in LD with SNPs in peaks and in DA peaks
Supplementary Table 54: List of IHEC datasets used for comparison with data generated in this study.

Supplementary Figures

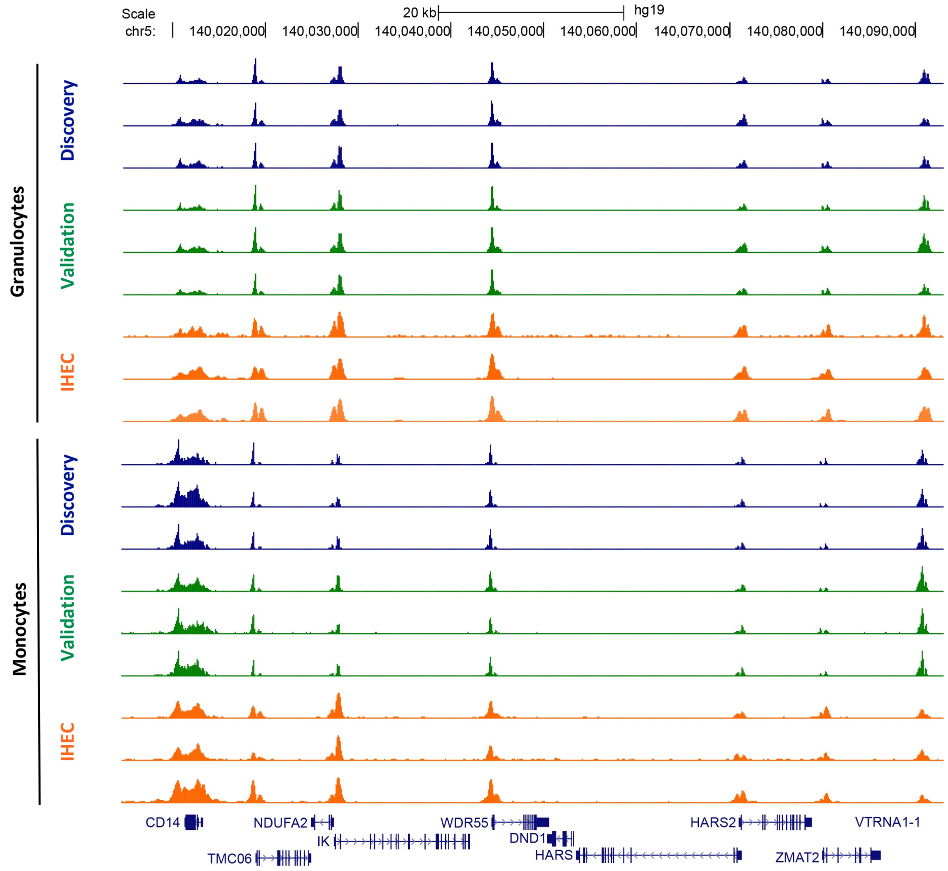

Supplementary Figure 1. Comparison of a locus with International Human Epigenomics Consortium (IHEC) H3K27ac ChIP-seq. UCSC Genome Browser view of a genomic locus: comparison of granulocyte and monocyte H3K27ac ChIP-seq profiles from discovery and validation cohorts with corresponding data from IHEC.

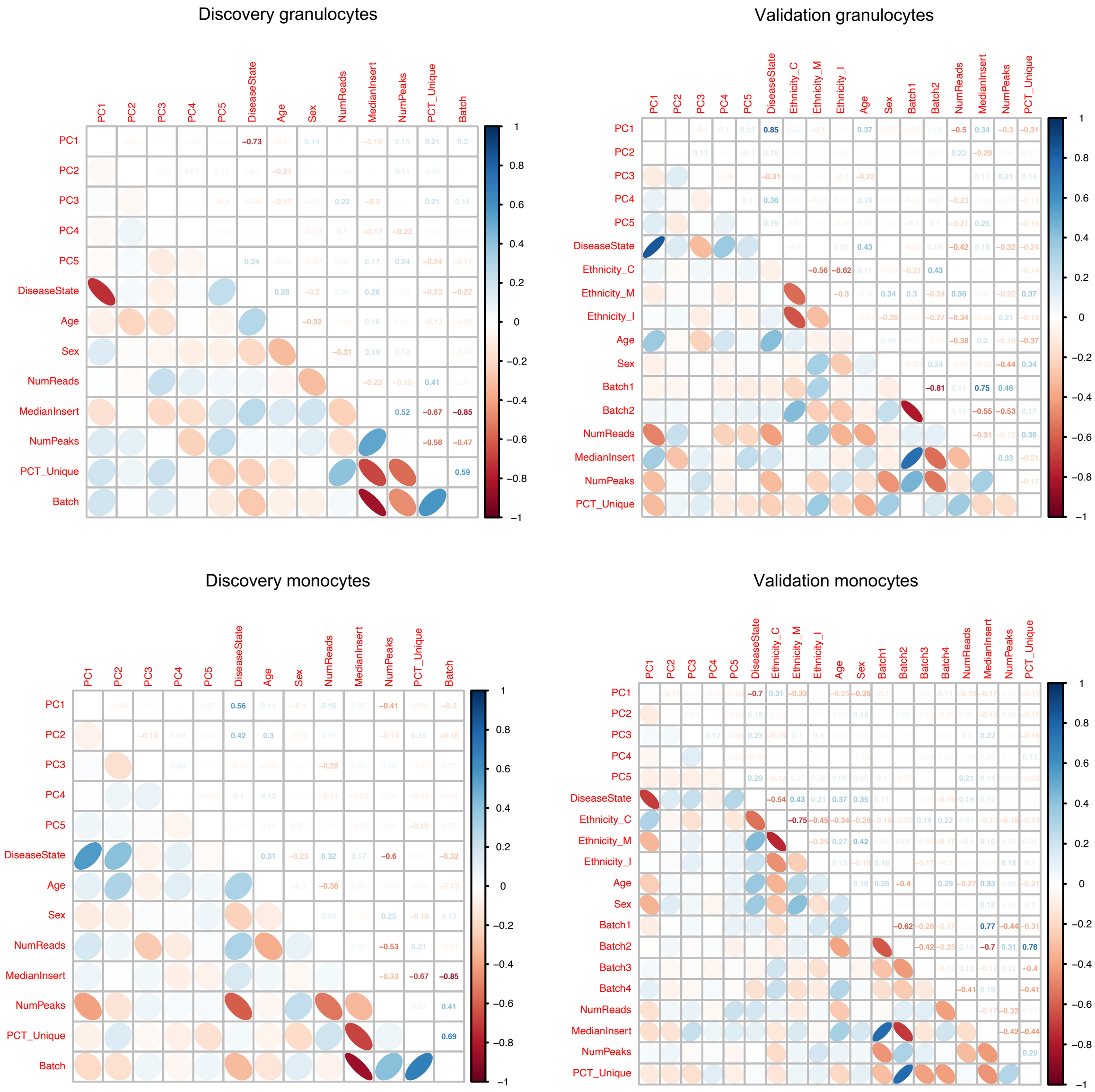

Supplementary Figure 2. Correlation among the first 5 Principal Components (PCs) of the residual peak height matrix and all biological and technical covariates in H3K27ac ChIP-seq data, discovery (Cohort 1) and validation (Cohort 2) cohorts. This is a symmetric matrix and the eccentricity of the ellipses and the intensity of the color are scaled to the correlation value. Blue ellipses: positive correlation, red ellipses: negative correlation.

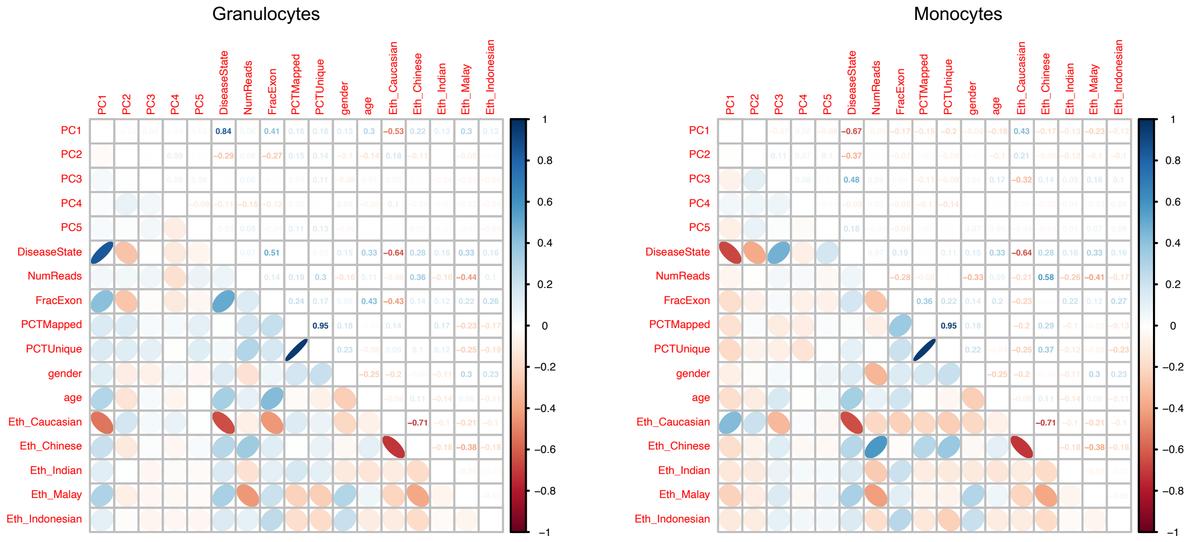

Supplementary Figure 3. Correlation among the first 5 principal Components (PCs) of the residual RNA-seq gene expression matrix and all biological and technical covariates (Cohort 3). This is a symmetric matrix and the eccentricity of the ellipses and the intensity of the color are scaled to the correlation value. Blue ellipses: positive correlation, red ellipses: negative correlation.

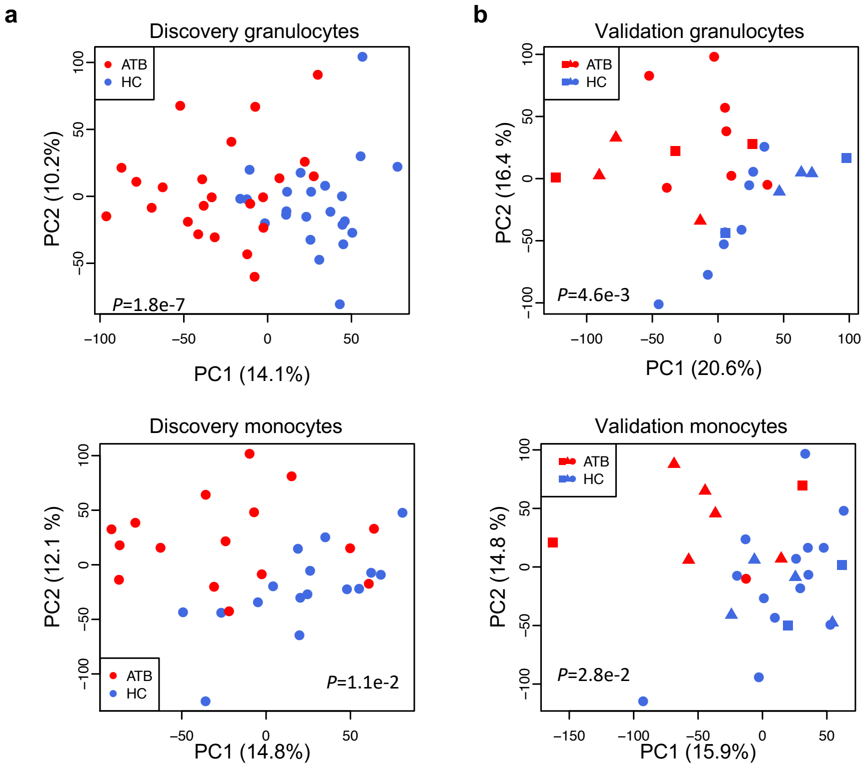

Supplementary Figure 4. PCA of granulocyte and monocyte peak heights in discovery (Cohort 1) and validation cohort (Cohort 2) using all peaks. **(a)** discovery cohort **(b)**, validation cohort (squares: Indian; circles: Chinese; triangles: Malay). *P*-values are from two-sided t-test of ATB vs HC PC1 values

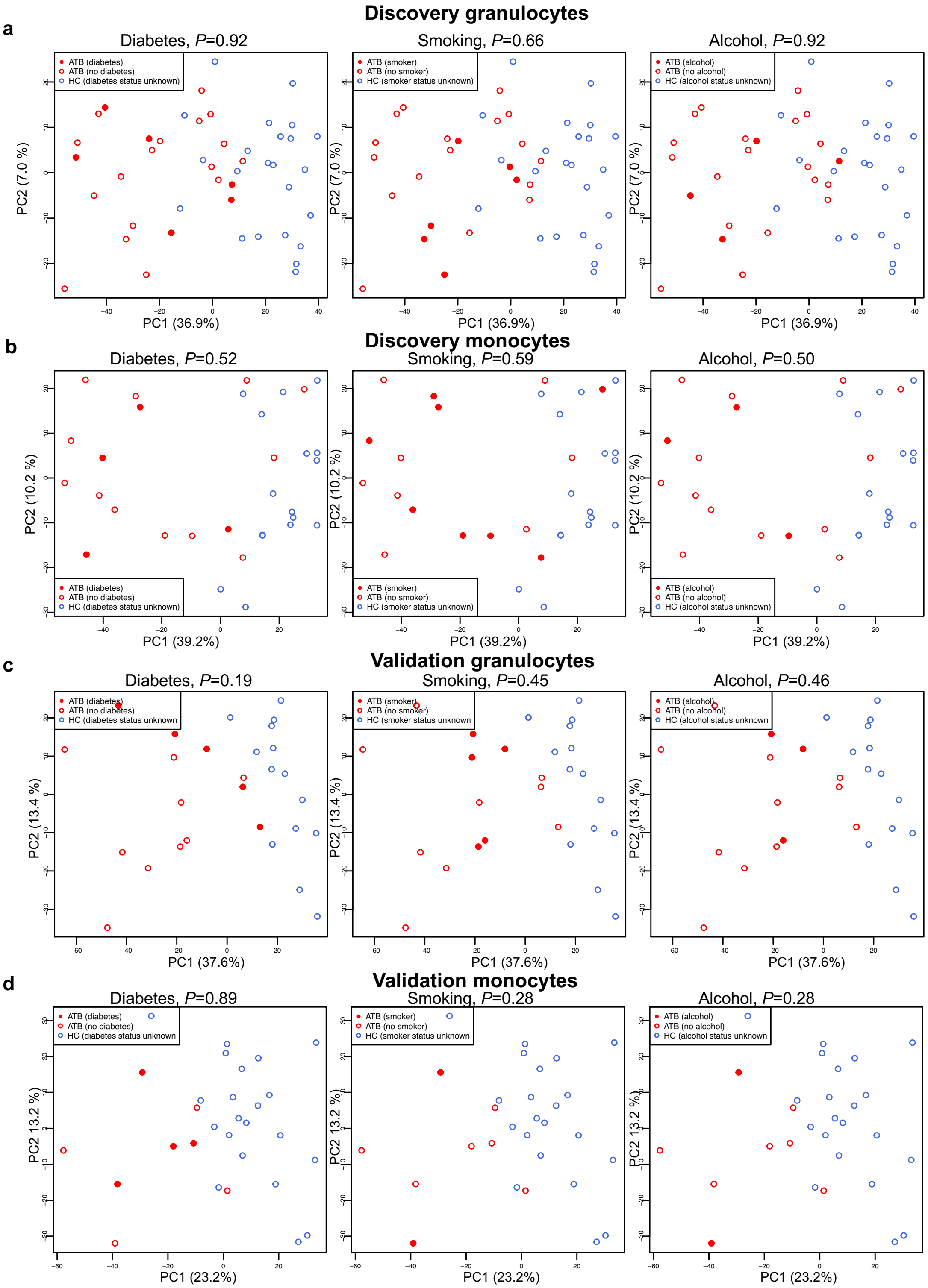

Supplementary Figure 5. PCA of granulocyte and monocyte peak heights in discovery (Cohort 1) and validation (Cohort 2) cohorts. Potential confounding factors (diabetes, smoking and alcohol) are indicated using filled circles. *P*-values are from two-sided t-test of solid red dots (ATB with condition) vs open red circles (ATB no condition) PC1 values.

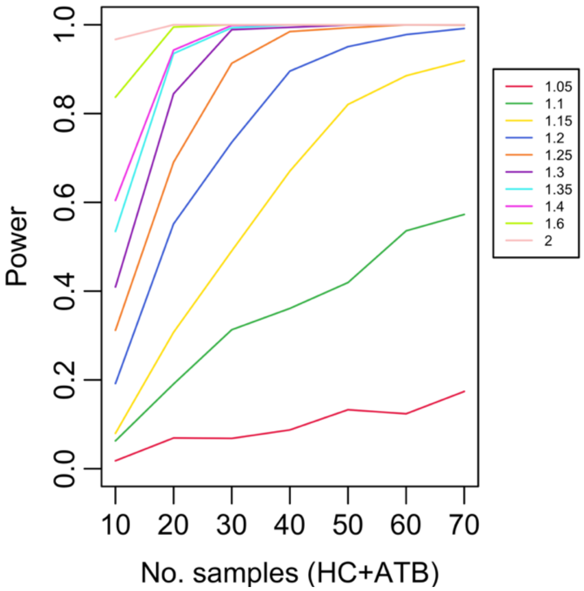

Supplementary Figure 6. Statistical power of DA peak calling as a function of peak height fold change and cohort size, estimated using simulated DA peaks.

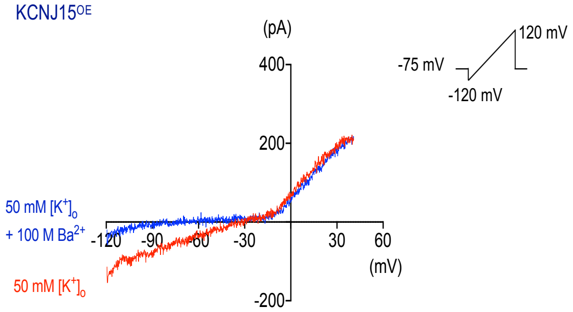

Supplementary Figure 7. Inwardly rectifying K^+ currents detected in KCNJ15^{OE} THP-1 cells. Patch clamp recordings of a KCNJ15^{OE} THP-1 cell using a ramp protocol from -120 mV to 120 mV in 200 ms showing amplitude of inwardly rectifying current inhibited by 0.1 mM Ba^{2+}.

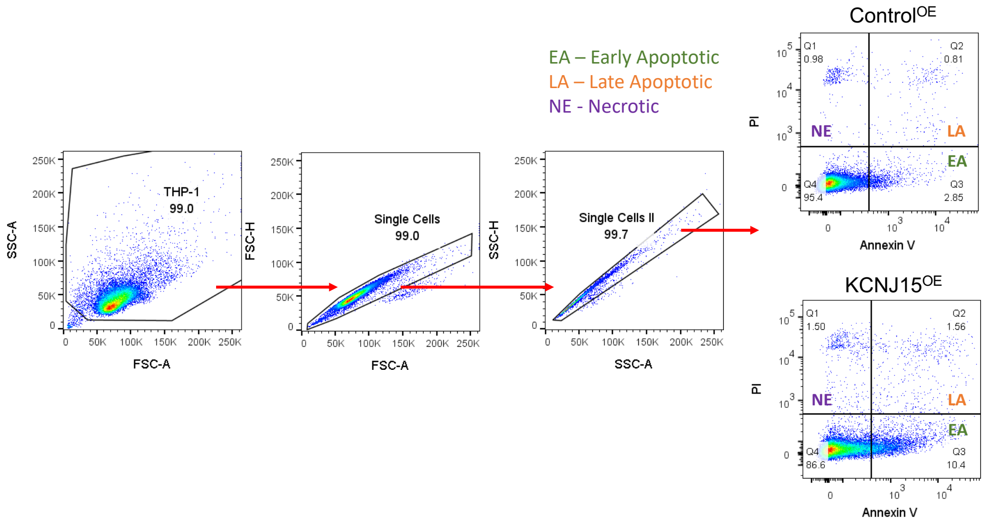

Supplementary Figure 8. Gating strategy to assess apoptosis in Control^{OE} and KCNJ15^{OE} THP-1 cells. Cells were stained with Annexin-FITC and PI (Methods) and were acquired on LSR Fortessa™, followed by analysis using FlowJo.

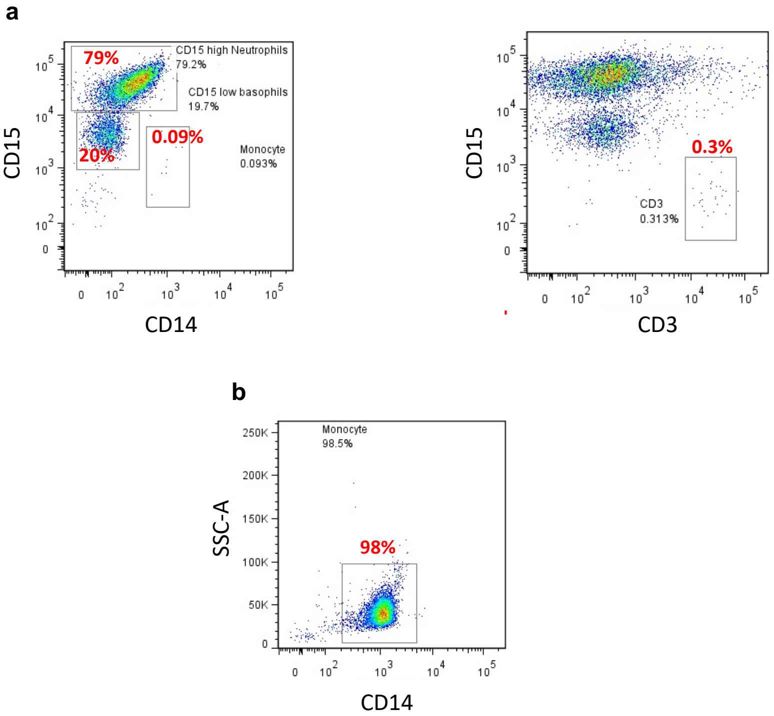

Supplementary Figure 9. Flow cytometric validation of purity of sorted cells from PBMCs. (**a**) Purity of granulocyte cell populations. (**b**) Purity of monocyte cell populations. Antibodies: anti-CD15-FITC, anti-CD14-APC, and anti-CD3-PECy7.
