# Supplementary material for: Histone acetylome-wide associations in immune cells from individuals with active Mycobacterium tuberculosis infection
Source: Nat Microbiol. 2022 Jan 31;7(2):312–26. doi: 10.1038/s41564-021-01049-w (PMC9439955; doi:10.1038/s41564-021-01049-w)
Supplement: Supplementary file 2 — Reporting Summary [file 41564_2021_1049_MOESM2_ESM.pdf]

nature research

Corresponding author(s): Shyam Prabhakar and Amit Singhal

Last updated by author(s): 18/11/21

Reporting Summary

Nature Research wishes to improve the reproducibility of the work that we publish. This form provides structure for consistency and transparency in reporting. For further information on Nature Research policies, see Authors & Referees and the Editorial Policy Checklist.

Statistics

For all statistical analyses, confirm that the following items are present in the figure legend, table legend, main text, or Methods section.

n/a	Confirmed	
☐	☒	The exact sample size (*n*) for each experimental group/condition, given as a discrete number and unit of measurement
☐	☒	A statement on whether measurements were taken from distinct samples or whether the same sample was measured repeatedly
☐	☒	The statistical test(s) used AND whether they are one- or two-sided<br>*Only common tests should be described solely by name; describe more complex techniques in the Methods section.*
☐	☒	A description of all covariates tested
☐	☒	A description of any assumptions or corrections, such as tests of normality and adjustment for multiple comparisons
☐	☒	A full description of the statistical parameters including central tendency (e.g. means) or other basic estimates (e.g. regression coefficient) AND variation (e.g. standard deviation) or associated estimates of uncertainty (e.g. confidence intervals)
☐	☒	For null hypothesis testing, the test statistic (e.g. *F*, *t*, *r*) with confidence intervals, effect sizes, degrees of freedom and *P* value noted<br>*Give P values as exact values whenever suitable.*
☒	☐	For Bayesian analysis, information on the choice of priors and Markov chain Monte Carlo settings
☒	☐	For hierarchical and complex designs, identification of the appropriate level for tests and full reporting of outcomes
☐	☒	Estimates of effect sizes (e.g. Cohen's *d*, Pearson's *r*), indicating how they were calculated

Our web collection on statistics for biologists contains articles on many of the points above.

Software and code

Policy information about availability of computer code

Data collection	No software was used for data collection
Data analysis	SAMtools (Li, 2011) version 0.1.19-44428cd<br>BWA (Li and Durbin, 2009) version 0.7.10-r789<br>Picard Tools broadinstitute.github.io/picard/<br>BedTools (Quinland and Hall 2010) version 2.25.0<br>DFilter (Kumar et al., 2013) version 1.6 http://collaborations.gis.a-star.edu.sg/~cmb6/kumarv1/dfilter/<br>GREAT (McLean et al., 2010) version 3.0.0<br>GORilla (Eden et al., 2009) http://cbl-gorilla.cs.technion.ac.il<br>HOMER (Heinz et al., 2010) version 4.10<br>GATK version 3.2-2 (DePristo et al., 2011) https://software.broadinstitute.org/gatk/<br>G-SCI test (del Rosario et al., 2015) http://collaborations.gis.a-star.edu.sg/~cmb6/G-SCI_test/<br>R The R Project for Statistical Computing https://www.r-project.org/<br>UCSC Genome Browser University of California Santa Cruz https://genome.ucsc.edu/<br>FlowJo Three Star https://www.flowjo.com/<br>ZEN software platform Carl Zeiss https://www.zeiss.com/microscopy/int/products/microscope-software/zen.html<br>softWoRX Suite 2.0 Applied Precision, GE Healthcare http://www.gelifesciences.co.kr/wp-content/uploads/2016/11/softworx_suite_overview.pdf<br>Imaris software (Andor-Bitplane, Zurich) Andor-Bitplane, Zurich www.bitplane.com/<br>LD score regression (Finucane, et al., 2015) https://github.com/bulik/ldsc

For manuscripts utilizing custom algorithms or software that are central to the research but not yet described in published literature, software must be made available to editors/reviewers. We strongly encourage code deposition in a community repository (e.g. GitHub). See the Nature Research guidelines for submitting code & software for further information.

Data

Policy information about availability of data

All manuscripts must include a data availability statement. This statement should provide the following information, where applicable:

- Accession codes, unique identifiers, or web links for publicly available datasets
- A list of figures that have associated raw data
- A description of any restrictions on data availability

ChIP-seq data have been deposited at the European Genome-phenome Archive EGA, http://www.ebi.ac.uk/ega/), which is hosted by the EBI, under accession number EGAS00001003118. RNA-seq data have been deposited at NCBI's Gene Expression Omnibus through GEO Series accession number GSE126614. The following figures have associated raw data provided as excel files: Figs. 1-4 and 6.

Field-specific reporting

Please select the one below that is the best fit for your research. If you are not sure, read the appropriate sections before making your selection.

☒ Life sciences ☐ Behavioural & social sciences ☐ Ecological, evolutionary & environmental sciences

For a reference copy of the document with all sections, see nature.com/documents/nr-reporting-summary-flat.pdf

Life sciences study design

All studies must disclose on these points even when the disclosure is negative.

Sample size	238 ChIP-seq, 78 RNA-seq
Data exclusions	For ChIP-seq samples, we discarded samples with low complexity as measured by the number of unique read-pairs (<15 million for Batches 1 and 2; <30 million for Batch 3). We also discarded samples with high GC bias (>4,500 peaks showing greater than two-fold GC bias in either direction). For RNA-seq samples, we discarded one sample with poor quality (percent reads mapped =45.3% and percent uniquely mapped reads=43.1%).
Replication	To ensure replication we performed a replication study (for ChIP-seq only)
Randomization	Covariates were controlled for by regression (see methods)
Blinding	The study was undertaken to find the H3K27ac and transcriptomic differences between known tuberculosis and healthy individuals. Hence no blinding was performed

Reporting for specific materials, systems and methods

We require information from authors about some types of materials, experimental systems and methods used in many studies. Here, indicate whether each material, system or method listed is relevant to your study. If you are not sure if a list item applies to your research, read the appropriate section before selecting a response.

Materials & experimental systems

n/a	Involved in the study	
☐	☒	Antibodies
☐	☒	Eukaryotic cell lines
☒	☐	Palaeontology
☒	☐	Animals and other organisms
☐	☒	Human research participants
☒	☐	Clinical data

Methods

n/a	Involved in the study	
☐	☒	ChIP-seq
☐	☒	Flow cytometry
☒	☐	MRI-based neuroimaging

Antibodies

Antibodies used	Anti-KCNJ15 (Sigma-Aldrich, #HPA016702)<br>anti-APAF-1 (Cell Signaling Technology, #5088)<br>anti-beta-actin (Cell Signaling Technology, #4967)<br>Annexin V APC (Biolegend, #640932)<br>anti–glyceraldehyde-3-phosphate dehydrogenase (GAPDH) (14C10) (Cell Signaling Technology, #2118)<br>anti-rabbit immunoglobulin G (IgG) horseradish peroxidase (HRP)-linked antibody (Cell Signaling Technology, #7074)<br>mouse IgG1 kappa isotype control eFluor 450 (eBioscience, #48-4714-82)<br>H3K27ac antibody for all ChIP experiments (Catalogue #39133; Active Motif)

Validation	Each antibody is validated for FACS staining as per manufacturers description. We also used FMO and isotype controls for validating the staining.

Eukaryotic cell lines

Policy information about cell lines

Cell line source(s)	Human monocyte THP-1 cells from ATTC
Authentication	This cell line was bought from ATCC
Mycoplasma contamination	This cell line was tested for Mycoplasma and was found to be negative.
Commonly misidentified lines (See ICLAC register)	*Name any commonly misidentified cell lines used in the study and provide a rationale for their use.*

Human research participants

Policy information about studies involving human research participants

Population characteristics	See Supplementary Table 1 and Supplementary Table 19
Recruitment	Singaporean Cohort (Cohorts 1-3):<br>HIV-negative ATB patients (based on clinical diagnosis with mycobacterial and radiographic evidence) and HC individuals (negative for IFN-γ release assay (IGRA); QuantiFERON TB gold test) were recruited at Tan Tock Seng Hospital's Tuberculosis Control Unit (TTSH, TBCU). Additional HC individuals were recruited locally at SIgN. Patients were sampled within 4 days of anti-TB treatment initiation and excluded if they had previously received anti-TB therapy. All participants provided written informed consent.<br><br>South African Cohort (Cohort 4):<br>1) ATB: All ATB patients (median age: 27.3; interquartile range (IQR): 23-33; 60% male) were tested sputum Xpert MTB/RIF (Xpert, Cepheid, Sunnyvale, CA) positive and had clinical symptoms and/or radiographic evidence of TB. All ATB cases were drug sensitive and had received no more than one dose of anti-tubercular treatment at the time of baseline blood sampling. A follow-up sample was obtained at completion of TB treatment (24 weeks).<br>2) LTBI: All individuals with latent Mtb infection (median age: 26.5; IQR: 23-33; 60% male) were asymptomatic, had a positive IFN-γ release assay (IGRA, QuantiFERON®-TB Gold In-Tube, Qiagen, Hilden), tested sputum Xpert MTB/RIF negative and exhibited no clinical evidence of active TB.<br>3) HC: Health controls (medium age: 30.5; IQR: 24-32; 40% male) were negative for IFN-γ release assay, tested sputum Xpert MTB/RIF negative and exhibited no clinical evidence of active TB.<br><br>In all cohorts, no compensation was given to any participant.
Ethics oversight	This study was approved by the Domain Specific Review Board of the National Healthcare Group (#2010/00566) and Institutional Review Board of the National University of Singapore (#09-256).<br>The South African cohort (Cohort 4) was recruited from the Ubuntu Clinic, Site B, Khayelitsha (Cape Town, South Africa) between March 2017 and December 2018. All participants were HIV-uninfected adults (age ≥ 18 yr) and provided written informed consent. The study was approved by the University of Cape Town Human Research Ethics Committee (HREC: 050/2015) and was conducted under DMID protocol no.15-0047.

Note that full information on the approval of the study protocol must also be provided in the manuscript.

ChIP-seq

Data deposition

☒ Confirm that both raw and final processed data have been deposited in a public database such as GEO.

☒ Confirm that you have deposited or provided access to graph files (e.g. BED files) for the called peaks.

Data access links<br>*May remain private before publication.*	EGAS00001003118 (EGA)
Files in database submission	T1G3_CON_dedupped.bam<br>T1G4_CON_dedupped.bam<br>T1G5_CON_dedupped.bam<br>T2G1_INF_dedupped.bam<br>T2G2_CON_dedupped.bam<br>T2G4_CON_dedupped.bam<br>T2G5_INF_dedupped.bam<br>T3G2_INF_dedupped.bam<br>T3G5_INF_dedupped.bam<br>T3G6_INF_dedupped.bam

T4G2_INF_dedupped.bam
T4G5_CON_dedupped.bam
T9G1_INF_dedupped.bam
T10G1_CON_dedupped.bam
T10G3_INF_dedupped.bam
T10G4_INF_dedupped.bam
T11G1_CON_dedupped.bam
T11G2_INF_dedupped.bam
T11G3_CON_dedupped.bam
T11G4_CON_dedupped.bam
T12G4_INF_dedupped.bam
T12G6_INF_dedupped.bam
T13G3_INF_dedupped.bam
T13G5_INF_dedupped.bam
T13G6_INF_dedupped.bam
T14G1_CON_dedupped.bam
T14G4_INF_dedupped.bam
CHP049_CON_dedupped.bam
CHP050_CON_dedupped.bam
CHP052_INF_dedupped.bam
CHP053_INF_dedupped.bam
CHP055_INF_dedupped.bam
CHP056_CON_dedupped.bam
CHP057_INF_dedupped.bam
CHP058_CON_dedupped.bam
CHP059_CON_dedupped.bam
CHP091_CON_dedupped.bam
CHP092_INF_dedupped.bam
CHP094_CON_dedupped.bam
CHP095_CON_dedupped.bam
CHP097_CON_dedupped.bam
CHP098_INF_dedupped.bam
CHP099_CON_dedupped.bam
CHP100_CON_dedupped.bam
CHP101_CON_dedupped.bam
T1G1_INF_dedupped.bam
T1G6_CON_dedupped.bam
T2G6_INF_dedupped.bam
T3G1_CON_dedupped.bam
T3G4_INF_dedupped.bam
T4G3_INF_dedupped.bam
T4G4_INF_dedupped.bam
T4G6_CON_dedupped.bam
T9G2_CON_dedupped.bam
T9G3_CON_dedupped.bam
T9G4_CON_dedupped.bam
T9G5_INF_dedupped.bam
T10G2_CON_dedupped.bam
T10G5_CON_dedupped.bam
T10G6_CON_dedupped.bam
T11G5_INF_dedupped.bam
T11G6_CON_dedupped.bam
T12G1_INF_dedupped.bam
T13G2_INF_dedupped.bam
T13G4_INF_dedupped.bam
T14G3_CON_dedupped.bam
CHP048_CON_dedupped.bam
CHP051_INF_dedupped.bam
CHP054_INF_dedupped.bam
CHP090_INF_dedupped.bam
CHP096_CON_dedupped.bam
CHP118_CON_dedupped.bam
CHP119_INF_dedupped.bam
T5M3_CON_dedupped.bam
T5M4_INF_dedupped.bam
T5M5_CON_dedupped.bam
T6M1_INF_dedupped.bam
T6M2_CON_dedupped.bam
T6M4_INF_dedupped.bam
T7M2_INF_dedupped.bam
T7M5_INF_dedupped.bam
T8M1_INF_dedupped.bam
T8M2_INF_dedupped.bam
T8M4_CON_dedupped.bam
T8M5_INF_dedupped.bam

T8M6_INF_dedupped.bam
CHP060_CON_dedupped.bam
CHP064_CON_dedupped.bam
CHP067_CON_dedupped.bam
CHP068_INF_dedupped.bam
CHP069_INF_dedupped.bam
CHP070_CON_dedupped.bam
CHP071_CON_dedupped.bam
CHP072_INF_dedupped.bam
CHP073_INF_dedupped.bam
CHP074_CON_dedupped.bam
CHP076_INF_dedupped.bam
CHP077_CON_dedupped.bam
CHP079_CON_dedupped.bam
CHP080_INF_dedupped.bam
CHP081_INF_dedupped.bam
CHP082_CON_dedupped.bam
CHP087_CON_dedupped.bam
CHP088_CON_dedupped.bam
CHP108_CON_dedupped.bam
T5M2_CON_dedupped.bam
T5M6_CON_dedupped.bam
T6M3_INF_dedupped.bam
T6M5_CON_dedupped.bam
T6M6_CON_dedupped.bam
T7M1_CON_dedupped.bam
T7M4_INF_dedupped.bam
T7M6_INF_dedupped.bam
T8M3_CON_dedupped.bam
CHP061_CON_dedupped.bam
CHP065_INF_dedupped.bam
CHP066_INF_dedupped.bam
CHP075_CON_dedupped.bam
CHP078_CON_dedupped.bam
CHP084_CON_dedupped.bam
CHP089_CON_dedupped.bam
CHP102_CON_dedupped.bam
CHP103_CON_dedupped.bam
CHP106_CON_dedupped.bam
CHP107_CON_dedupped.bam
CHP110_CON_dedupped.bam
CHP111_CON_dedupped.bam
CHP112_INF_dedupped.bam
CHP113_INF_dedupped.bam
CHP126_CON_dedupped.bam
CHP127_CON_dedupped.bam
CHP129_CON_dedupped.bam
CHP130_INF_dedupped.bam
CHP132_CON_dedupped.bam
TB__1_trimmed_dedup.bam
TB__13_trimmed_dedup.bam
TB__14_trimmed_dedup.bam
TB__16_trimmed_dedup.bam
TB__17_trimmed_dedup.bam
TB__18_trimmed_dedup.bam
TB__19_trimmed_dedup.bam
TB__20_trimmed_dedup.bam
TB__22_trimmed_dedup.bam
TB__23_trimmed_dedup.bam
TB__24_trimmed_dedup.bam
TB__25_trimmed_dedup.bam
TB__26_trimmed_dedup.bam
TB__3_trimmed_dedup.bam
TB__30_trimmed_dedup.bam
TB__32_trimmed_dedup.bam
TB__34_trimmed_dedup.bam
TB__36_trimmed_dedup.bam
TB__37_trimmed_dedup.bam
TB__38_trimmed_dedup.bam
TB__39_trimmed_dedup.bam
TB__4_trimmed_dedup.bam
TB__41_trimmed_dedup.bam
TB__5_trimmed_dedup.bam
TB__6_trimmed_dedup.bam

TB__7_trimmed_dedup.bam

Genome browser session (e.g. UCSC) | no longer applicable

Methodology

Replicates: Each sample was sequenced once.

Sequencing depth: The reads are paired end 2x100 bp. For each sample, the number of non-redundant read-pairs (min 16.8 million read pairs, average 52.6 million read pairs) and the median insert size are given in Supplementary Tables 2, 4, 6, 8 and 19.

Antibodies: H3K27ac antibody Catalogue #39133; Active Motif

Peak calling parameters:

Alignment: bwa mem -t 4 hg19.fa read1.fastq read2.fastq | samtools view -Sbh - > bamfile.bam

convert bam to bed using bedTools: bamToBed -i bamfile.bam 1>bamtobed.bed

Peak Calling: run_dfilter.sh -d=bamtobed.bed -c=bamtobed_input.bed -o=outputfile -ks=100 -redund=1000

Data quality: Duplicate reads (read-pairs mapping to the same genomic location) were collapsed. For each sample, ChIP-seq peaks were detected using DFilter at a P-value threshold of 1e-6. For either cell type, the initial peak set was defined as the union of peaks from the entire set of discovery and validation samples from Batches 1 and 2, which included the vast majority of samples (Table S1). Peaks wider than 8Kb were then discarded. We performed multi-sample correction for GC bias (refs 13, 37) separately on each processing batch. We then discarded samples with low complexity as measured by the number of unique read-pairs (<15 million for Batches 1 and 2; <30 million for Batch 3). We also discarded samples with high GC bias (>4,500 peaks showing greater than two-fold GC bias in either direction). The number of peaks called for each sample is given in Supplementary Tables 2, 4, 6, and 8. Coordinates (bed format) of DA peaks are in Supplementary Tables 3, 5, 7 and 9.

Software:
read mapping: bwa 0.7.10-r789, samtools 0.1.19-44428cd
bedtools: bedtools v2.25.0
peak calling: DFilter1.6

Flow Cytometry

Plots

Confirm that:

- [x] The axis labels state the marker and fluorochrome used (e.g. CD4-FITC).
- [x] The axis scales are clearly visible. Include numbers along axes only for bottom left plot of group (a 'group' is an analysis of identical markers).
- [ ] All plots are contour plots with outliers or pseudocolor plots.
- [x] A numerical value for number of cells or percentage (with statistics) is provided.

Methodology

Sample preparation: PBMCs and granulocytes were isolated from freshly drawn blood. From PBMCs, monocytes were isolated using CD14+ immunomagnetic separation beads (MACS, Miltenyi). Isolated CD14+ and granulocytes were assessed for purity by flowcytometry. In some experiments monocytes were stimulated with potassium for defined time-point. Cells (unstimulated and stimulated) were washed and single-cell suspensions were resuspended in PBS and stained for 30min with live/dead fixable aqua dead cell stain kit (Thermo Fisher Scientific, #L34965). Cells were then stained with respective antibodies. In experiments where phosporylated substrate was assessed, Live/Dead stained cells were washed with FACS buffer (containing PBS with 3% FBS, 1mM EDTA and 0.1% sodium azide) and incubated with Human TruStain FcX™ (BioLegend, #422302) for 20min. Following this, cells were washed with FACS buffer, fixed and permeabilized using the PerFix EXPOSE kit (Beckman Coulter, #B26976) according to manufacturer's instructions. Permeabilized cells were incubated for 30 min in the dark with fluorophore-conjugated antibodies specific for phosphorylated marker or isotype control antibodies.

Instrument: BD LSRII

Software: BD FACSDiva software was used to collect the acquired flow data. Data was analysed using FlowJo software.

Cell population abundance: The purified CD14+ monocytes and granulocytes showed purity >95%.

Gating strategy: The CD14+ mononuclear cells were FSC/SSC gated. Upon this single cells were gated using FSC-H/FSC-A. Single cells were used to gate out dead cells using fixable live-dead stain. Live cells were assessed for the staining by antibodies targeting phosphorylated substrates.
Gating strategy for apoptosis experiment has been added in Supplementary Fig 8

- [x] Tick this box to confirm that a figure exemplifying the gating strategy is provided in the Supplementary Information.
