# Supplementary figures and images for: Histone acetylome-wide associations in immune cells from individuals with active Mycobacterium tuberculosis infection
Source: Nat Microbiol. 2022 Jan 31;7(2):312–26. doi: 10.1038/s41564-021-01049-w (PMC9439955; doi:10.1038/s41564-021-01049-w)

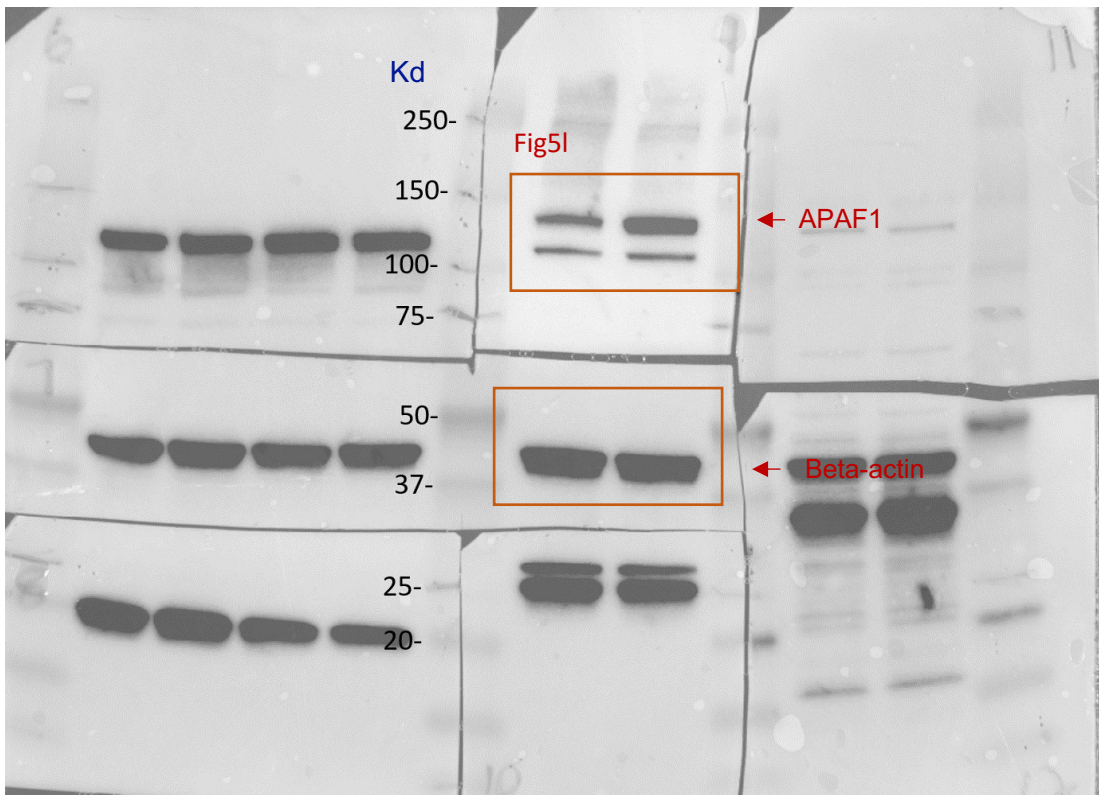

Supplement: Source Data Fig. 5 — Unprocessed blots. [file 41564_2021_1049_MOESM11_ESM.pdf]
