# Supplementary material for: Histone acetylome-wide associations in immune cells from individuals with active Mycobacterium tuberculosis infection
Source: Nat Microbiol. 2022 Jan 31;7(2):312–26. doi: 10.1038/s41564-021-01049-w (PMC9439955; doi:10.1038/s41564-021-01049-w)
Supplement: Source Data Extended Data Fig. 4 — Unprocessed blots. [file 41564_2021_1049_MOESM17_ESM.pdf]

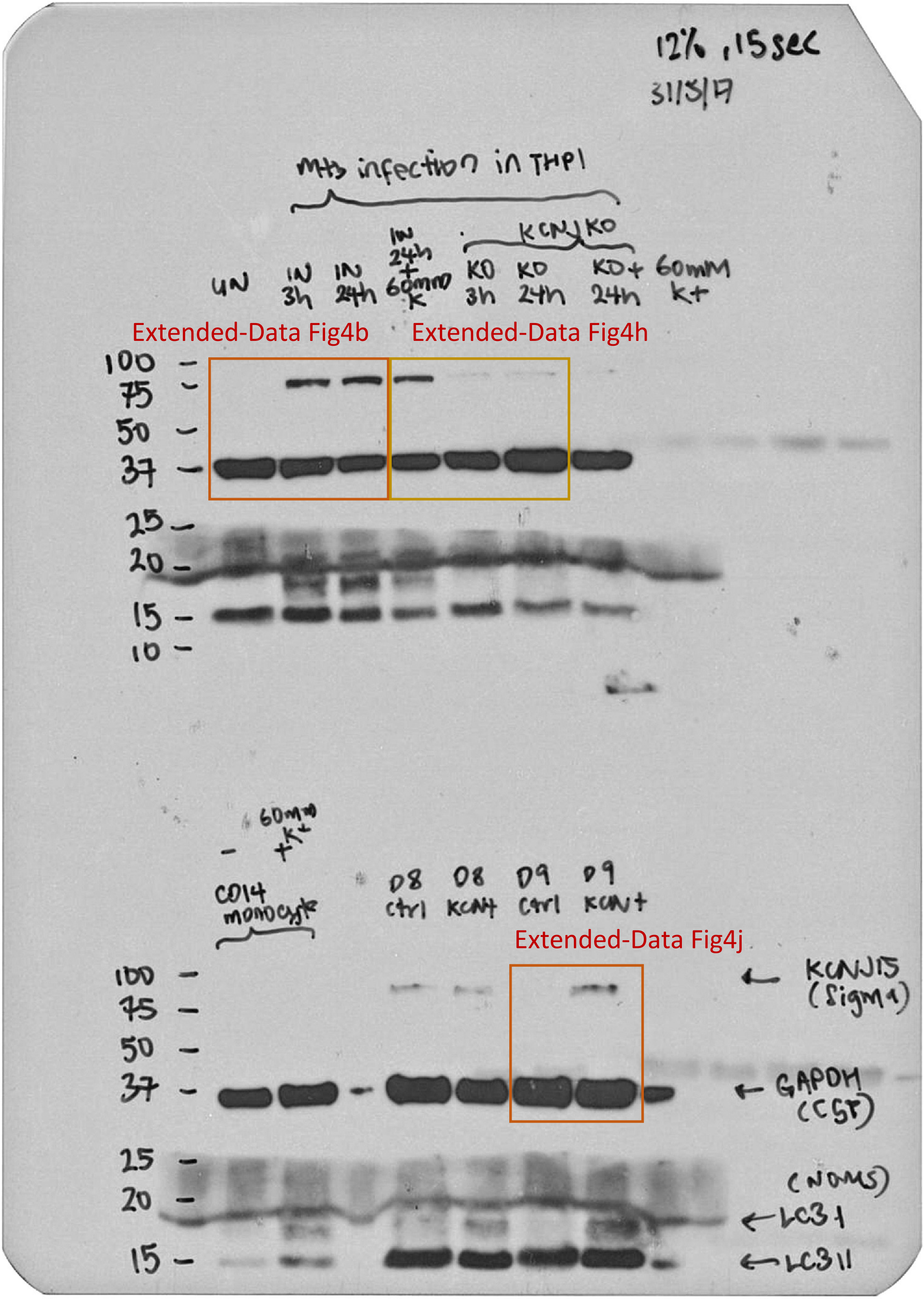
12% ,15sec
31/5/17
Mtb infection in THP1
KCNJ KO
UN
IN 3h
IN 24h
IN 24h + 60mm K
KO 3h
KO 24h
KO+ 24h
60mM K+
Extended-Data Fig4b
Extended-Data Fig4h
100
75
50
37
25
20
15
10
60mm K+
CD14 monocyte
D8 ctrl
D8 KCN+
D9 ctrl
D9 KCN+
Extended-Data Fig4j
KCNJ15 (Sigma)
GAPDH (CST)
(NOMS)
LC3 I
LC3 II
